# EEG signatures of cognitive decline after mild SARS-CoV-2 infection: an age-dependent study

**DOI:** 10.1186/s12916-024-03481-1

**Published:** 2024-06-20

**Authors:** Yike Sun, Jingnan Sun, Xiaogang Chen, Yijun Wang, Xiaorong Gao

**Affiliations:** 1 The School of Biomedical Engineering, Tsinghua University, Beijing, 100084 China; 2 Institute of Biomedical Engineering, Chinese Academy of Medical Sciences and Peking Union Medical College, Tianjin, 300192 China; 3 Institute of Semiconductor, Chinese Academy of Sciences, Beijing, 100083 China

**Keywords:** SARS-CoV-2, EEG, Mild illness, Cognitive impact, Long COVID

## Abstract

**Background:**

Current research on the neurological impact of SARS-CoV-2 primarily focuses on the elderly or severely ill individuals. This study aims to explore the diverse neurological consequences of SARS-CoV-2 infection, with a particular focus on mildly affected children and adolescents.

**Methods:**

A cohort study was conducted to collect pre- and post-infection resting-state electroencephalogram (EEG) data from 185 participants and 181 structured questionnaires of long-term symptoms across four distinct age groups. The goal was to comprehensively evaluate the impact of SARS-CoV-2 infection on these different age demographics. The study analyzed EEG changes of SARS-CoV-2 by potential biomarkers across age groups using both spatial and temporal approaches.

**Results:**

Spatial analysis indicated that children and adolescents exhibit smaller changes in brain network and microstate patterns post-infection, implying a milder cognitive impact. Sequential linear analyses showed that SARS-CoV-2 infection is associated with a marked rise in low-complexity, synchronized neural activity within low-frequency EEG bands. This is evidenced by a significant increase in Hjorth activity within the theta band and Hjorth mobility in the delta band. Sequential nonlinear analysis indicated a significant reduction in the Hurst exponent across all age groups, pointing to increased chaos and complexity within the cognitive system following infection. Furthermore, linear regression analysis based on questionnaires established a significant positive relationship between the magnitude of changes in these neural indicators and the persistence of long-term symptoms post-infection.

**Conclusions:**

The findings underscore the enduring neurological impacts of SARS-CoV-2 infection, marked by cognitive decline and increased EEG disarray. Although children and adolescents experienced milder effects, cognitive decline and heightened low-frequency electrical activity were evident. These observations might contribute to understanding potential anxiety, insomnia, and neurodevelopmental implications.

## Background

The eruption of the SARS-CoV-2 pandemic has instigated a global public health crisis, posing significant threats to respiratory health [1–3]. Significantly, this crisis has not only posed a substantial menace to the respiratory system [4, 5] but has also sparked concerns regarding its impact on the central nervous system [6–8]. A wealth of empirical research has confirmed that SARS-CoV-2 can induce a range of neurological issues, notably affecting cognitive functions [9, 10]. Amidst various methodologies employed for cognitive function assessment, electroencephalography (EEG) techniques emerge as pivotal tools [11] for evaluating cognitive function and quantifying the detrimental effects of SARS-CoV-2 infection on cognitive performance [12, 13].

However, existing research predominantly focuses on EEG studies involving elderly and severely affected patients [10, 14–16]. Recent shifts in focus explore the effects on younger, more diverse populations. For instance, in 2024, researchers employed EEG to analyze sleep patterns in children post-SARS-CoV-2 infection [17]. Although numerous comparative EEG studies have targeted younger demographics [18–20], these investigations often involve limited participant numbers and age ranges. Therefore, it is critical to expand EEG studies to more comprehensively assess the long-term cognitive impacts of SARS-CoV-2.

The primary aim of this study is to bridge the gap in understanding the cognitive effects of SARS-CoV-2 in individuals presenting mild symptoms, with a focus on EEG patterns across different age groups, especially in children and adolescents. We gathered resting EEG data from a diverse cohort of 185 individuals who experienced mild symptoms related to SARS-CoV-2, both before infection and after full recovery. Utilizing advanced analytical techniques such as source connectivity and microstate analysis, this study explores the subtle cognitive changes induced by SARS-CoV-2, analyzing both spatial and temporal aspects.

Against the backdrop of the globally reported tally of more than 770 million confirmed cases of SARS-CoV-2 infection as of September 29, 2023 [21], it is of paramount importance to fathom the cognitive implications wrought by SARS-CoV-2 infection upon the substantial proportion of individuals who exhibit mild symptoms. Such an endeavor is indispensable not only for enhanced comprehension of the virus itself but also for the formulation of healthcare strategies and support systems, with a specific focus on the child and adolescent demographics alongside other vulnerable segments of the population. Our investigation serves to elucidate the intricacies surrounding the cognitive ramifications of SARS-CoV-2 infection in mildly symptomatic populations across varying age groups, thereby contributing to the foundation of rehabilitation strategies geared towards ameliorating the afflictions of SARS-CoV-2 and mitigating the challenges posed by long COVID or post-COVID-19 syndrome [22].

## Methods

### Study design

The data elucidated in this investigation emanate from a comprehensive longitudinal EEG study, tracking EEG recordings across diverse age cohorts. Initially, the scope of the research was not aligned with clinical objectives. Nevertheless, an unforeseen opportunity arose due to a pivotal shift in China’s public health policy after 2022. Consequentially, a significant proportion of the participants contracted the SARS-CoV-2 virus within a markedly narrow timeframe—specifically, not exceeding a 1-week variance—and uniformly achieved recovery within 4 weeks. All participants in the study were clinically classified as having mild manifestations of the disease and were experiencing their first infection. This unique circumstance allowed us to capture and analyze the EEG data from these individuals’ pre-infection and post-recovery, providing an invaluable comparative perspective on the neurophysiological impact of SARS-CoV-2.

EEG recordings before infection were taken 1 to 2 months before the participants tested positive for SARS-CoV-2 via nasal or throat swab tests. Follow-up EEG recordings were performed 1 to 2 months after the participants tested negative. During the data collection phases, participants were placed in a controlled environment—a small, brightly-lit room devoid of any visual stimuli that might influence the EEG results. Participants were instructed to remain seated, avoid bodily or eye movements, and keep their eyes open throughout the recording session.

### Participants

This study was enhanced by administering a structured questionnaire to a group of 181 participants, consisting of 88 males and 93 females. The data curation and validation process yielded 185 reliable EEG recordings after excluding data affected by noise or interference. It is essential to note that the subset of participants providing EEG data did not completely overlap with those responding to the questionnaire. The participants were divided into four age categories: child (under 10 years), adolescent (10 to 20 years), young adult (20 to 27 years), and adult (over 27 years), with group sizes of 63, 28, 39, and 55, respectively. All subjects had prior exposure to long-term EEG studies, which acquainted them with the EEG recording procedure. Consequently, sequential effects were minimized in this study, though they could not be entirely disregarded.

For the EEG analysis, the groups included adults (*n* = 55), young adults (*n* = 39), adolescents (*n* = 28), and children (*n* = 63). The mean age of the adult group was 31.64 years (SD = 5.61), comprising 58% females; the young adult group had a mean age of 24.36 years (SD = 1.48), with 77% females; the adolescent group’s mean age was 15.07 years (SD = 1.03), with 29% females; and the child group’s mean age was 7.49 years (SD = 1.47), with 30% females. All participants resided in North China, were diagnosed with mild clinical conditions, and had no neurological lesions attributed to SARS-CoV-2 (Table 1).

**Table 1:** Basic information of the population in this study

Group	Age	Num of EEG	Num of questionnaire
Adult	[26, 52)	55	54
Young adult	[20, 25]	39	41
Adolescent	[10, 19]	28	26
Child	[4, 9]	63	60

### EEG data preprocessing

The EEG dataset analyzed in this research encompasses eye-open resting-state data acquired using a saline electrode device recorded at a sampling rate of 100 Hz (JBZH-16–1, BRAINNEWLIFE, 16-channel system). All lead positions are arranged according to the 10–20 standard. A preprocessing protocol was implemented to maintain data integrity. During the recording phase, a specialist flagged any segments where significant body movements caused electrode dislodgement. To mitigate the impact of ocular and muscular artifacts, the independent component analysis (ICA) was employed. Additionally, direct current (DC) and instrumental frequency (IF) interferences were eliminated using a bandpass filter ranging from 0.5 to 45 Hz. These preprocessing steps were critical to ensuring the reliability and validity of the study’s findings.

In the analytical phase, the EEG data was segregated into six distinct frequency bands using a specialized filter bank, covering the full frequency spectrum: full band (0.5–45 Hz), delta (0.5–4 Hz), theta (4–8 Hz), alpha (8–12 Hz), beta (12–30 Hz), and gamma (30–45 Hz). An averaging reference operation was subsequently applied across all datasets to ensure analytical consistency and accuracy.

### Brain network source connectivity analysis in the spatial domain

Source connectivity analysis is a critical technique using neuroimaging data for examining complex interactions between brain regions [23]. Its main goal is to identify functional or effective linkages among cerebral sources that reflect cognitive shifts in conditions like depression and schizophrenia [24, 25]. Among various functional connectivity metrics, coherence is a key measure, calculating the linear correlation between two signals in the frequency domain. However, coherence measurements can be affected by volumetric conduction, causing misleading pseudo-coherent values [26, 27].

In response, several effective connectivity measures, such as the directional transfer function (DTF) and partially directional coherence (PDC), have been proposed [28]. The direct directed transfer function (dDTF), a modification of the DTF method, is especially notable. It incorporates Granger causality principles and allows distinguishing between direct and indirect connections [29]. This study employs the dDTF method for source linkage analysis.

In this research, we initially computed the connectivity data from EEG recordings taken prior to infection as well as from data collected post-infection and during recovery. These results were then subjected to statistical testing. Ultimately, we highlighted findings demonstrating statistically significant reductions, along with their respective differences, within the study.

### Microstate analysis in the spatial domain

EEG microstate analysis is a key methodology in neuroscience, providing deep insights into spontaneous brain activity [30]. It assumes EEG stability over short time periods, segments EEG signals into brief, stable scalp electrical topographies, and uses cluster analysis to reveal potential functional changes [31–33]. Microstate analysis is widely used in diverse neuroscience studies, including examinations of brain states in neuropsychiatric disorders and normative aging and developmental processes [34, 35]. This study applies the KMeans clustering method for microstate analysis of EEG data and uses the MNE-Python toolkit for visualizing microstate topographic data [36]. It is important to note, however, that due to variations in sampling frequency and timing in this study compared to most other studies, the resulting microstate topography maps differ. Nevertheless, since this study focuses on comparing the relative relationship between pre- and post-intervention states, this discrepancy is considered justifiable.

### Linear analysis in time sequence

For an in-depth analysis of temporal variation in EEG signals, we have chosen the Hjorth parameter and Kolmogorov complexity as key metrics. The Hjorth parameter, commonly used in EEG analysis and epilepsy detection studies, consists of three elements: activity (HA), mobility (HM), and complexity (HC) [37–39]. HA measures signal power, reflecting brain activity or arousal levels. HM quantifies mean frequency, providing insights into the synchronization of brain activity and dynamic neural processes. HC gauges frequency change in an EEG signal, reflecting the regularity or irregularity of brain activity [40].

Kolmogorov complexity, on the other hand, examines the shortest algorithmic length of a string [41]. In our analysis, it is used to characterize the minimum representation length of an EEG signal, with higher values indicating more intricate activity [42, 43]. To compute Kolmogorov complexity, we used a threshold at 0 for binarization, enabling an effective evaluation of complexity metrics in the EEG data.

### Nonlinear analysis in time sequence

Our methodology uses nonlinear approaches to assess EEG signal changes pre- and post-SARS-CoV-2 infection. Sample entropy, an improvement over approximate entropy, measures time series complexity and pattern generation likelihood [44–46]. Due to its computational independence from data length and enhanced consistency, it becomes a robust measure for assessing EEG’s nonlinear processes [47, 48].

The Hurst index identifies the long-term memory of a time series, providing insight into brain activity and function [49]. It also highlights differences in EEG signals across brain regions, age groups, and mental states [50]. We use detrended fluctuation analysis (DFA) to calculate the Hurst index, which effectively eliminates potential spurious long-range correlations due to the non-smoothness of temporal order in EEG signals, revealing intrinsic long-range correlations in complex systems [51].

### Statistical test methods

In this study, statistical analyses were conducted to ensure the robustness and validity of the findings. First and foremost, normality testing was performed using the Shapiro–Wilk normality test, a fundamental step in validating the assumptions underlying parametric statistical methods. Subsequently, for datasets adhering to a normal distribution (*P* > 0.05), a paired *t*-test was employed, a method widely acknowledged for its appropriateness in comparing means under normal conditions. In cases where the data did not conform to a normal distribution (*P* ≤ 0.05), the analysis was conducted using the Wilcoxon signed-rank test, a non-parametric test known for its effectiveness in evaluating differences between paired samples without relying on the assumptions of normality. All statistical procedures were executed with the SCIPY.STATS toolkit for Python.

## Results

### Spatial biomarkers: source connectivity analysis

**Fig. 1:**
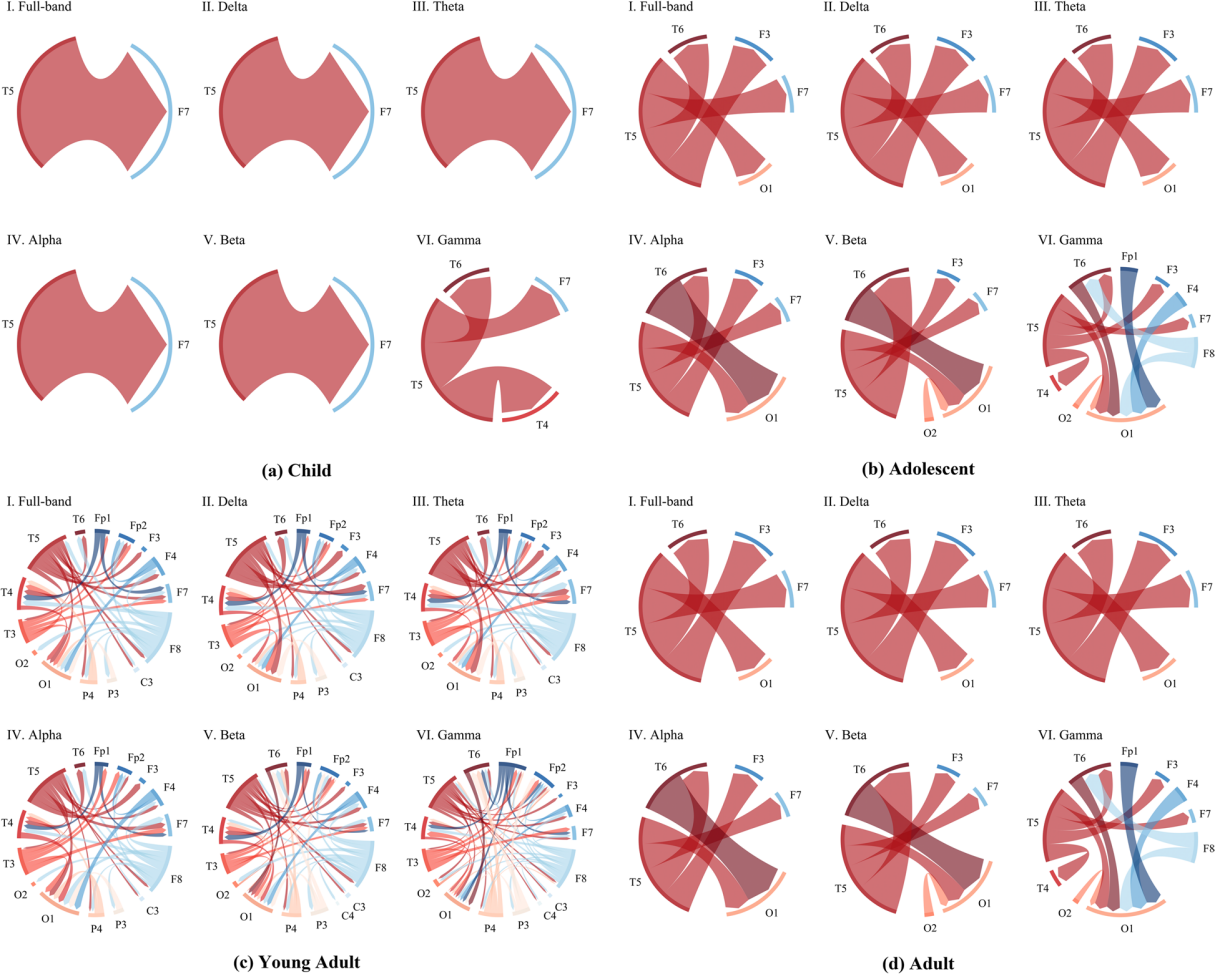
This figure displays chordal plots representing brain network source connectivity analysis outcomes. The colors in the plots designate outgoing source leads: red for T-region, orange for O-region, pink for P-region, blue for F-region, and dark blue for FP-region. Each subplot (**a** to **d**) represents different age groups: child, adolescent, young adult, and adult. Subplots I to VI depict results across various frequency bands: full, delta, theta, alpha, beta, and gamma

To evaluate the impact of SARS-CoV-2 infection on cognitive processes over time, we conducted a source connectivity analysis using EEG data, collected before infection and after recovery. We employed the dDTF, known for its effectiveness in reducing signal interference caused by the volumetric conductor effect in EEG studies. Our analysis revealed statistically significant reductions in connectivity, as illustrated in Fig. 1. Notably, the reduction in connectivity was particularly evident around the T5 region, which is closely linked to memory, language, and emotion processing. Previous studies have suggested that decreased connectivity in this region is associated with cognitive changes observed in conditions such as attention deficit hyperactivity disorder (ADHD) and mild cognitive impairment (MCI) [52, 53]. Our findings suggest that SARS-CoV-2 infection could potentially lead to noticeable cognitive decline.

Moreover, the majority of the interactions showing significant declines were from the T-region to the F-region, which are areas typically associated with task execution and memory/decision-making, respectively. This pattern suggests that the infection may result in long-term deficits in cognitive and decision-making functions.

A key observation was that statistically significant reductions in connectivity were mainly intra-hemispheric (left in odd leads, right in even leads), indicating that the cognitive impact of SARS-CoV-2 might be limited in scope. However, the effects on higher cognitive functions appear more pronounced, as evidenced by significant decreases at higher frequencies.

Age-related differences in the impact of SARS-CoV-2 were also apparent. Young adults showed the most significant cognitive impact, followed by adults and adolescents, while children under 10 exhibited the least effect, with significantly fewer link reductions compared to young adults. These findings suggest that the cognitive resilience varies with age, with the brain networks of young adults being notably more vulnerable to disruption by SARS-CoV-2. This vulnerability could be influenced by factors such as the stage of brain development, lifestyle, or pre-existing health conditions. Adults and adolescents displayed moderate resilience, while the minimal impact on children could indicate more robust brain networks or compensatory mechanisms that protect against connectivity loss.

### Spatial biomarkers: microstate analysis

**Fig. 2:**
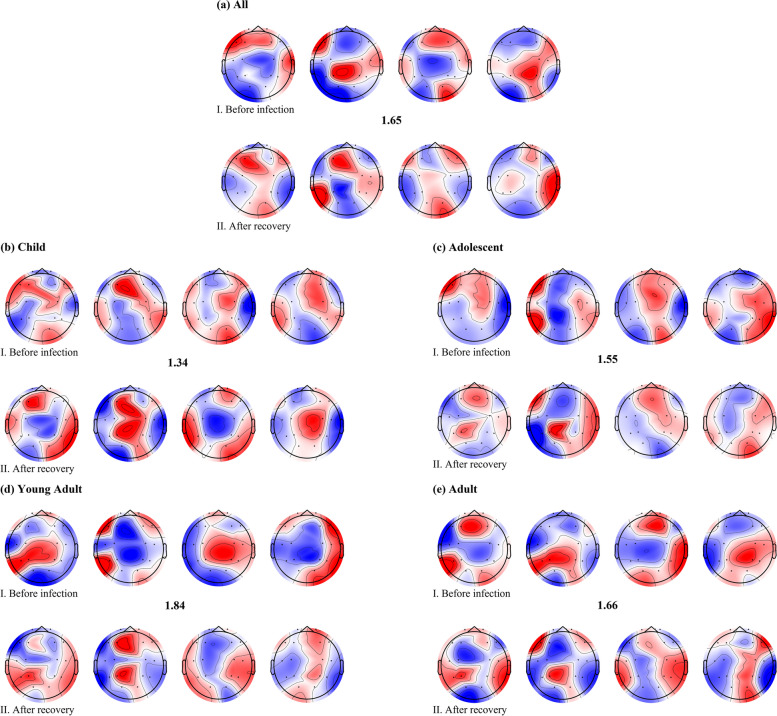
This figure illustrates the changes in microstate analysis before and after SARS-CoV-2 infection. Part I displays the four microstates before infection (arranged by decreasing frequency from left to right), while part II shows the results following recovery. The numerical values between parts I and II indicate the mean Euclidean distances for the four microstates pre- and post-infection. Specifically, **a** represents the clustered outcomes for the entire population, whereas **b**, **c**, **d**, and **e** show the results for the child, adolescent, young adult, and adult groups, respectively

A comprehensive clustering analysis of EEG microstates, conducted before and after SARS-CoV-2 infection, revealed distinct patterns. Following this, a distance analysis was performed on the central microstate patterns identified by the clustering, using Euclidean distance as the metric for differentiation. The results, along with the EEG topographies of these microstates, are presented in Fig. 2.

In the aggregate, the disparity in EEG microstates before and after infection manifested as 1.65. This deviation was discerned as 1.34 for the child group, 1.55 for the adolescent group, 1.84 for the young adult group, and 1.66 for the adult group. This numerical representation is posited to encapsulate the alteration in microstate patterns, with a higher deviation indicative of a more substantial shift in cognitive patterns. Benchmarking against the deviation value across all groups, it is discerned that the cognitive pattern alteration for the child and adolescent groups is below the population average. Conversely, the adult group exhibits a marginally higher cognitive pattern change, while the young adult group demonstrates the most considerable alteration, surpassing all other age cohorts.

This analysis leads us to conclude that the young adult group experienced the most substantial impact from SARS-CoV-2 infection, with the adult group also significantly affected. The child and adolescent groups, however, seemed to maintain more stable cognitive patterns post-infection.

It is important to note, however, that these results were not compared against a control group of uninfected individuals. Therefore, we cannot entirely exclude the potential influence of external factors such as social pressure. Unfortunately, it is now challenging to find uninfected control subjects for such studies. Therefore, these findings should be interpreted with caution. Nonetheless, given the short intervals between signal acquisitions, significant changes in cognitive patterns were unlikely. The minimal change observed in the fastest-developing child and adolescent groups further supports the notion that the adult and young adult groups were more significantly affected.

### Sequence biomarkers: linear analysis

In the analysis of EEG time sequences, our initial focus was on quantifying energy changes. However, these outcomes were omitted from the narrative due to the absence of statistically significant alterations in energy levels before and after SARS-CoV-2 infection. This absence of discernible energy shifts implies that the impact of SARS-CoV-2 on EEG may not attain a pathological magnitude, thereby implying that cognitive changes resulting from SARS-CoV-2 infection may not reach pathological thresholds. In light of this, we computed HA, HM, HC, and KC parameters.

**Fig. 3:**
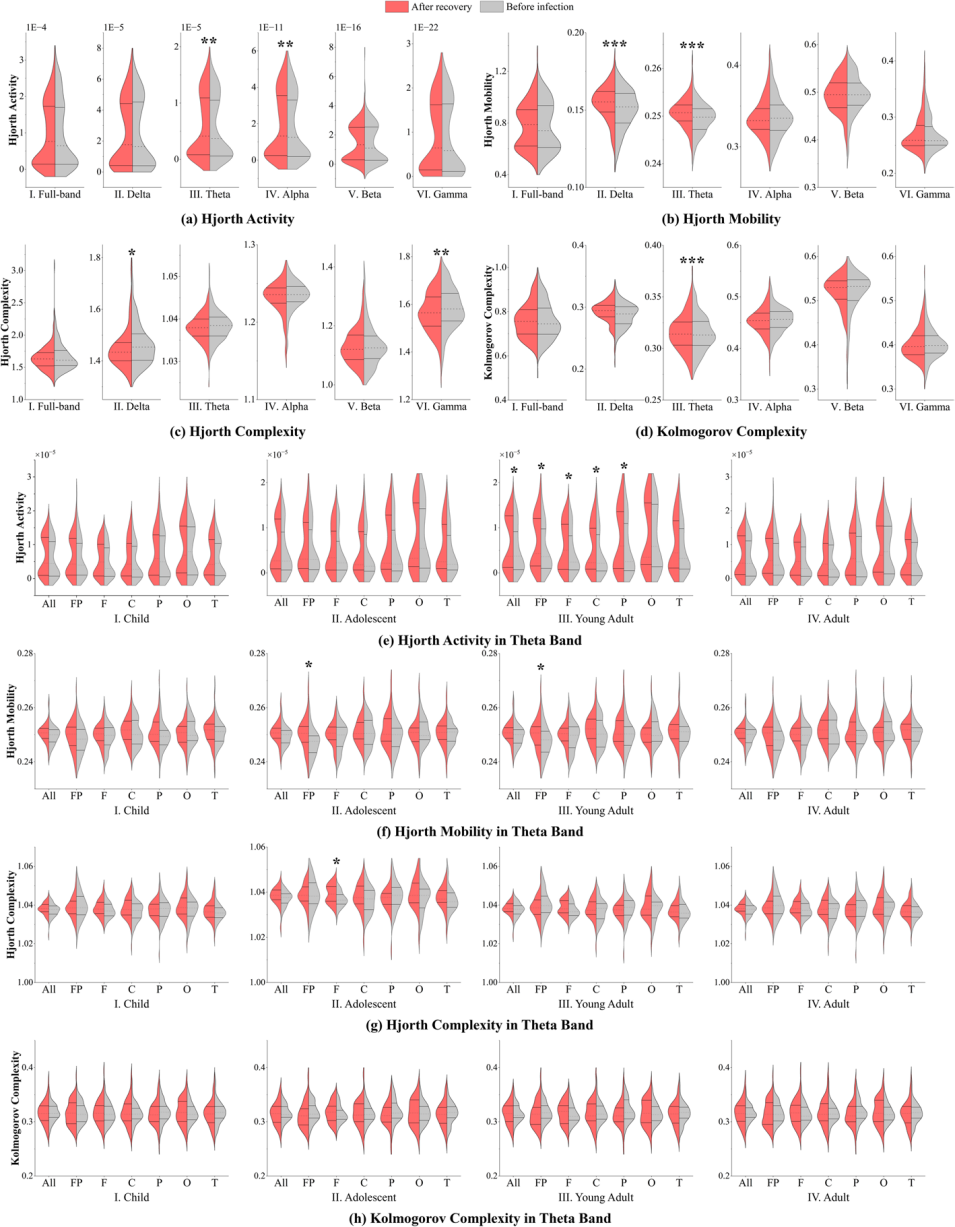
This figure presents bilateral violin plots illustrating the distribution of linear analysis sequence biomarkers across four age cohorts before infection and after recovery. **a** to **d** detail the outcomes associated with HA, HM, HC, and KC parameters, respectively, across all frequency bands: full, delta, theta, alpha, beta, and gamma. **e** to **h** focus on the theta band for each age group: child, adolescent, young adult, and adult. Each panel contrasts the pre-infection EEG parameters (gray area) against the post-recovery parameters (red area), with asterisks indicating statistically significant results

HA parameter intricately linked to EEG energy changes. As shown in Fig. 3a, our statistical analysis unveiled a noteworthy surge in theta (50.96 percentage points; 95% CI, − 316.53 to 418.46 percentage points; *P* = 0.0096 < 0.01) and alpha (52.84 percentage points; 95% CI, − 360.17 to 465.84 percentage points; *P* = 0.008 < 0.01) bands following recovery from SARS-CoV-2 infection. This compellingly indicates heightened EEG activation in theta and alpha bands across all demographics post-infection and recovery.

Upon this foundation, a thorough examination of the HM parameters in conjunction with the HC parameters was conducted, as depicted visually in Fig. 3b and c for each frequency band across all populations. The comparison between pre-infection EEG and post-recovery EEG reveals a discernible trend. Significantly, the HM parameters demonstrated statistically noteworthy elevations in both the delta band (3.15 percentage points; 95% CI, − 15.13 to 21.42 percentage points; *P* = 0.0001 < 0.001) and theta band (0.48 percentage points; 95% CI, − 2.49 to 3.44 percentage points; *P* = 0.000045 < 0.001) after SARS-CoV-2 infection. This finding implies that, following infection and subsequent recovery from SARS-CoV-2, the population displayed heightened alterations in frequency within the delta and theta bands, accompanied by a discernible degree of synchronization in brain activity.

Conversely, the analysis of HC parameters unveiled notable alterations in the delta band (− 0.93 percentage points; 95% CI, − 13.49 to 11.64 percentage points; *P* = 0.022 < 0.05) and gamma band (− 0.98 percentage points; 95% CI, − 10.13 to 8.17 percentage points; *P* = 0.0027 < 0.01) before and after infection. In stark contrast to the observed augmentation in HM parameters, the HC parameters exhibited a reduction. This discrepancy suggests that the EEG signal manifests a diminished rate of frequency change in both delta and gamma bands post-SARS-CoV-2 infection and recovery. Consequently, this distinction implies a diminished occurrence of perturbations and changes in the EEG signal following SARS-CoV-2 infection and recovery, accompanied by a decrease in the complexity of the time domain.

Having scrutinized the enhanced EEG activity through parametric analysis of HM and HC, a subsequent step involved binarizing the EEG signals and conducting a comprehensive analysis of signal activity complexity using the KC parameters, as illustrated in Fig. 3d. The resultant figure unequivocally demonstrates a substantial elevation in the KC parameter within the delta band post-SARS-CoV-2 infection compared to the pre-infection state (1.97 percentage points; 95% CI, − 11.48 to 15.42 percentage points; *P* = 0.00067 < 0.001). This observed phenomenon implies a noteworthy increase in the length of the shortest algorithm describing the EEG, indicative of heightened pattern changes in EEG dynamics after infection with SARS-CoV-2 and recovery.

From the findings of the aforementioned analysis, it is evident that following infection with and recovery from SARS-CoV-2, there is a discernible augmentation in the extent of low-complexity activity in the EEG. However, the overall complexity of the EEG registers a decline owing to the escalated prevalence of low-complexity activity, consequently resulting in an elevation of the HM parameter and a concomitant reduction in the HC parameter. The upsurge in the KC parameter signifies an augmentation in low-complexity synchronized activity that was nonexistent before the viral infection, constituting an entirely novel pattern of neural activity. We posit that the SARS-CoV-2 infection precipitates an influx of novel low-complexity synchronized activity in the EEG, reminiscent to some extent of the abnormal discharge activity observed in epilepsy, albeit with a considerably diminished degree of variability.

Our investigation unveils a notable concentration of alterations within the theta band, compelling an exploration of this specific frequency range. As depicted in Fig. 3e to h, a discernible trend in the association between HM, KC, and decreasing HC across all age groups and leads is evident.

For HA analysis in theta band, only the young adult group exhibits a simultaneous increase across the whole brain regions (58.71 percentage points; 95% CI, − 196.22 to 313.64 percentage points; *P* = 0.012 < 0.05), as well as in the prefrontal, frontal, central, and parietal areas. And the adult cohort manifested statistically significant variations in prefrontal zone leads (0.85 percentage points; 95% CI, − 4.65 to 6.35 percentage points; *P* = 0.045 < 0.05) concerning the HM parameter. Simultaneously, the adolescent cohort also exhibited significant alterations in prefrontal zone leads (1.45 percentage points; 95% CI, − 4.18 to 7.08 percentage points; *P* = 0.029 < 0.05). Furthermore, the child cohort and adult cohort did not exhibit statistical significance. Concerning the HC parameters, only the adolescent cohort exhibited a statistically significant alteration in frontal areas (0.25 percentage points; 95% CI, − 0.73 to 1.23 percentage points; *P* = 0.017 < 0.05). This alteration may imply the emergence of a greater number of novel EEG patterns within the occipital lobe region. Contrastingly, for the KC parameters, no significant changes were observed in any of the four age groups.

Notably, these findings substantiate the proposition that SARS-CoV-2 infection may impact perceptual awareness, with observed changes predominantly localized in the prefrontal and frontal region. In the aggregate, in terms of *P* values, the young adult cohort attained the highest significance, followed by the adolescent cohort, the adult cohort, and the child cohort in descending order. It is noteworthy that the changes in linear analysis sequence biomarkers attributable to SARS-CoV-2 infection were more conspicuous in the young adult and adult cohorts than in other cohorts.

### Sequence biomarkers: nonlinear analysis

**Fig. 4:**
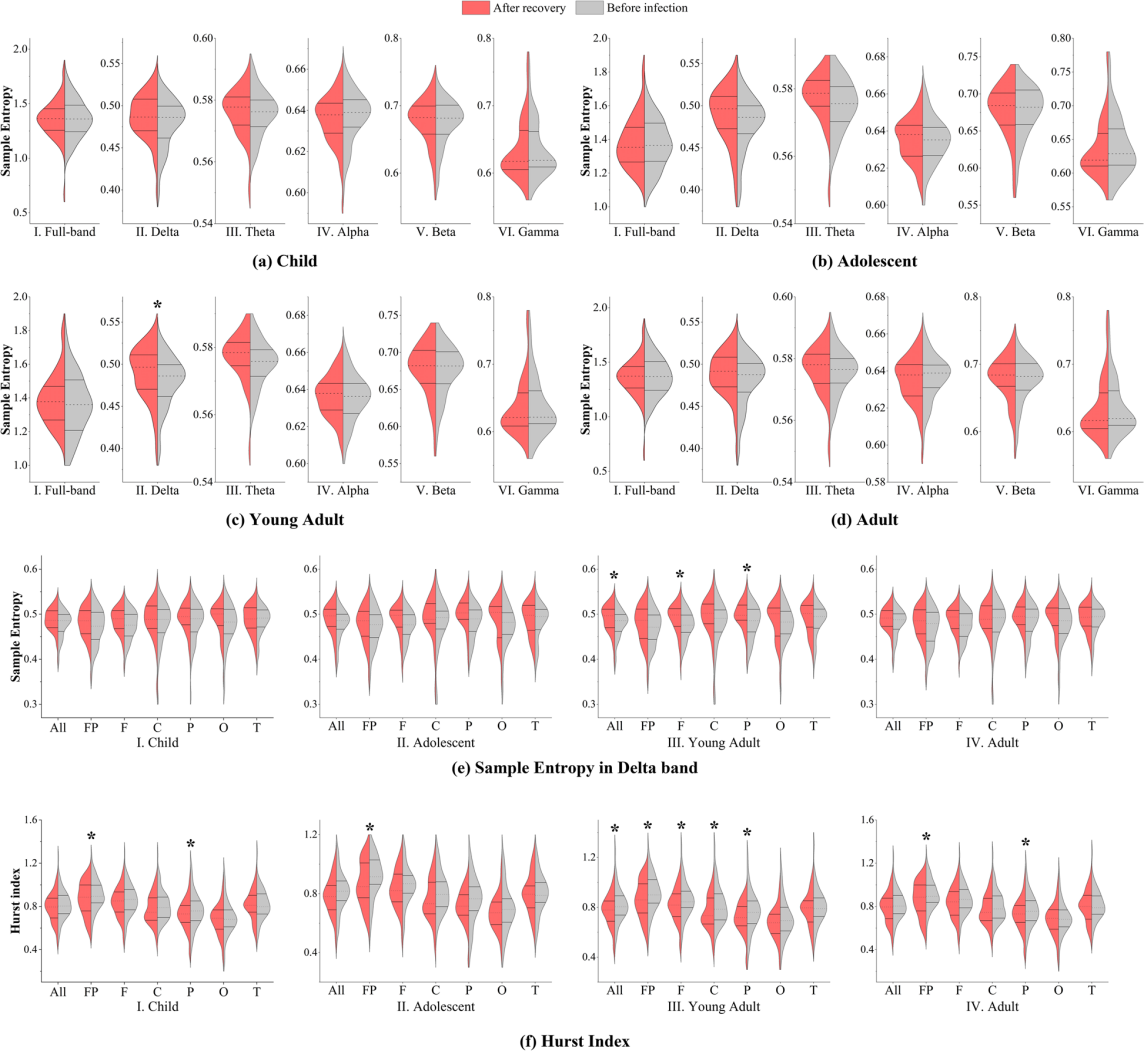
This figure presents bilateral violin plots illustrating the distribution of nonlinear analysis sequence biomarkers before infection and after recovery across four age cohorts. **a** through **d** display the sample entropy analysis results for the child, adolescent, young adult, and adult groups, respectively. Each panel details the outcomes for six frequency bands: full band, delta, theta, alpha, beta, and gamma. **e** provides a focused view of the sample entropy analysis within the delta band across different cerebral leads. **f** depicts the results of the Hurst index analysis conducted via the DFA method, detailing findings across all age groups. The labels ALL, FP, F, C, P, O, and T represent full-lead averaged results, and results for the prefrontal, frontal, central, parietal, occipital, and temporal areas, respectively

In the examination of nonlinearity, the initial step involved the computation of sample entropy, with the outcomes graphically depicted in Fig. 4a to d. In comparison to the pre-infection state, the sample entropy of EEG after SARS-CoV-2 infection and recovery demonstrated an ascending tendency. However, none of these alterations attained statistical significance, except for the young adult cohort, wherein a noteworthy increase in the delta band was observed (2.43 percentage points; 95% CI, − 11.01 to 15.86 percentage points; *P* = 0.027 < 0.05). This observation signifies a discernible augmentation in nonlinear activity within the delta frequency band among young adults. Notably, this frequency band is widely acknowledged for its association with the underlying neural processes of sleep and mood regulation. Consequently, the discerned escalation in the delta band suggests a predisposition of the young adult cohort to post-infection cognitive disorders related to sleep and mood.

The findings of the region-specific analysis for each age group are delineated in Fig. 4e to undertake a more granular examination of the variations within the delta frequency band. Evidently, within the young adult cohort, a statistically significant elevation in sample entropy is evident in both the frontal and parietal regions. These cerebral regions are integral to diverse cognitive functions, encompassing attention, memory, decision-making, and sensory integration. Consequently, this outcome posits a plausible influence of SARS-CoV-2 infection on attentional processes within the young adult demographic. The discerned effects were most pronounced within the young adult cohort, with relatively diminished impacts observed in the remaining age groups, particularly among children and adolescents.

We conducted a comprehensive examination of the Hurst index to scrutinize the long-term memory characteristics inherent in the EEG time series. The outcomes of this analysis, presented in Fig. 4f through a full band exploration, unveil noteworthy findings. Specifically, within the child cohort, a substantial reduction in the prefrontal (− 3.44 percentage points; 95% CI, − 34.25 to 27.37 percentage points; *P* = 0.034 < 0.05) and parietal (− 3.2 percentage points; 95% CI, − 41.01 to 34.61 percentage points; *P* = 0.028 < 0.05) regions is evident. Analogously, the adolescent group manifests a comparable noteworthy decrease in the prefrontal regions (− 5.03 percentage points; 95% CI, − 28.66 to 18.59 percentage points; *P* = 0.036 < 0.05). The young adult group exhibits a simultaneous decline across the whole brain regions (− 3.66 percentage points; 95% CI, − 28.43 to 21.12 percentage points; *P* = 0.028 < 0.05), as well as in the prefrontal (− 4.68 percentage points; 95% CI, − 31.14 to 21.79 percentage points; *P* = 0.013 < 0.05), frontal (− 3.55 percentage points; 95% CI, − 29.1 to 22 percentage points; *P* = 0.0296 < 0.05), central (− 5.78 percentage points; 95% CI, − 36.01 to 24.46 percentage points; *P* = 0.028 < 0.05), and parietal (− 3.59 percentage points; 95% CI, − 38.63 to 31.45 percentage points; *P* = 0.036 < 0.05) areas. In the case of the adult group, the reduction in significance extends to the prefrontal (− 4.31 percentage points; 95% CI, − 36.11 to 27.49 percentage points; *P* = 0.032 < 0.05) and parietal (− 4.38 percentage points; 95% CI, − 41.36 to 32.59 percentage points; *P* = 0.025 < 0.05) regions.

In interpreting the results, we observe a noticeable decline in the Hurst index following SARS-CoV-2 infection and subsequent recovery. This trend suggests a reduction in the long-term regularity of EEG signals, indicative of increased randomness in brain activity. However, it is crucial to consider that this decrease in the Hurst index might not solely reflect changes in cognitive processes. Factors such as alterations in cognitive function and variations in sleep–wake states, which are not directly measured in this study, could also influence these results. Therefore, while the data suggest an increase in the chaotic and complex nature of the cognitive system, potentially leading to higher anxiety levels, these interpretations should be approached with caution. The impact appears most pronounced in the young adult group, followed by adults, children, and adolescents, as inferred from the analysis of respective *P* values. Future studies should aim to disentangle the effects of cognitive and sleep–wake changes from those directly related to viral infection to better understand the mechanisms underlying these observations.

### Behavioral questionnaire results and regression analysis

**Fig. 5:**
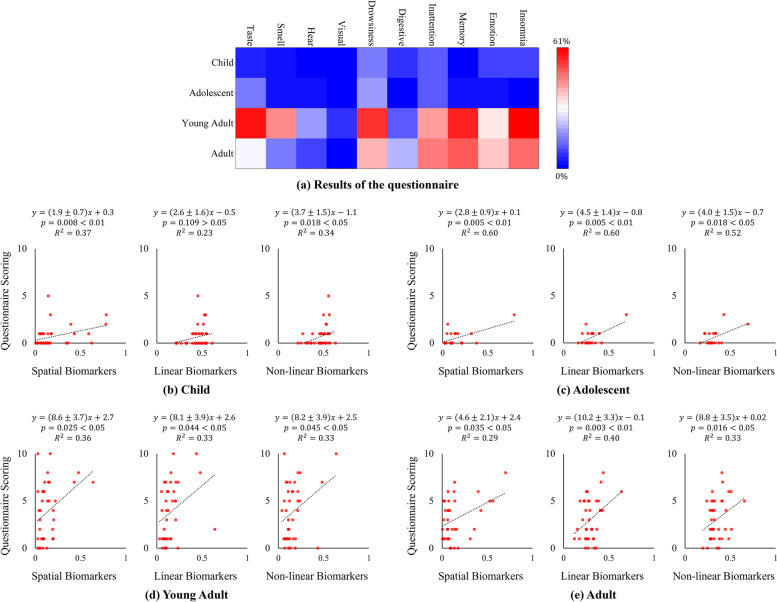
** a** shows the outcomes of the questionnaire through a heatmap. The graph’s horizontal axis represents four distinct age groups, while the vertical axis denotes potential symptoms relevant to cognition. The color intensity conveys the ratio of the number of people with specific symptoms to the total number of people within a given age group. Red hues signify a higher rate of occurrence, whereas blue indicates a lower rate of the corresponding symptom manifesting in that age group. **b** to **e** represent the results of regression analyses of spatial biomarkers, linear biomarkers, and nonlinear biomarkers against questionnaire scores for each of the four age groups, where the equation represents the expression of the fitted line

After acquiring the EEG data, a supplementary questionnaire was administered to the participants with the primary objective of scrutinizing potential cognitive symptoms such as insomnia, mood disorders, and memory impairments. The outcomes depicted in Fig. 5a reveal distinctive patterns among age groups. During the survey process, participants reported their symptoms, marking “1” if they perceived the symptom and “0” if they did not. Significantly, the young adult group demonstrated the highest prevalence of cognitive dysfunctions, closely followed by the adult cohort. In contrast, the adolescent and child groups showed a lower probability of exhibiting cognitive-related symptoms. This pattern is consistent with the insights obtained from the comprehensive analyses conducted previously.

To enhance the robustness of the association between the identified potential biomarkers and symptomatology delineated in the preceding analysis, we operationalized the questionnaire responses into discrete scores. Each of the ten symptoms enumerated was assigned a corresponding score based on participant responses, yielding an aggregate score with a potential range from 0 to 10. Subsequently, we normalized the transformation magnitude across the various biomarker indices to fall within a unified spectrum of 0 to 1 and computed their mean to ascertain the average biomarker alteration.

It is imperative to note that we calculated the change in biomarker levels as an absolute value, given that the correlation between these indicators and symptomatology is not presupposed to be linear. The regression analysis outcomes, depicted in Fig. 5b to e, illustrate our findings across four distinct age cohorts. It is evident from these results that—except for the child age group, where the link between the linear series of biomarkers and symptoms did not reach statistical significance—the remaining age groups exhibited a notable positive correlation. This correlation signifies that as the degree of deviation in the three categories of biomarkers escalates, there is a concomitant intensification of cognitive and psychiatric symptoms.

## Discussion

The outcomes of this study distinctly highlight the amplified susceptibility of young adults to cognitive deficits following a SARS-CoV-2 infection, a demographic that has traditionally not been considered as high risk. This is predominantly observable in the significant decrement in EEG source connectivity, particularly within the region of the temporal lobe, a key area for the functions of memory, language, and emotional processing. Such modifications could potentially result in cognitive deterioration, displaying patterns akin to those observed in cases of ADHD and MCI. Our findings propose a more profound impact of SARS-CoV-2 on young adults in comparison to adolescents and children. This insight can potentially steer the formulation of rehabilitation strategies tailored for long COVID patients.

The diminished connectivity in specific brain regions, such as electrode T5, which in temporal lobe, may reflect disruptions in neural networks that are crucial for cognitive functions [54]. This aligns with existing studies that link changes in brain connectivity to various cognitive impairments [55]. The persistence of connectivity reductions primarily within hemispheres further underscores the targeted impact of SARS-CoV-2 on brain function. The increase in the HA parameter within the theta band post-infection in adults suggests subtle yet discernible changes in EEG activity, potentially reflecting alterations in cognitive states. The heightened complexity in EEG patterns post-recovery, particularly in the delta band, might indicate a compensatory neural mechanism or an altered state of brain activity in response to the infection.

The observed concentration of alterations within the delta frequency band presents a pioneering insight, proposing that this band may be particularly susceptible to the neurological impacts of SARS-CoV-2 [56]. Traditionally, it is recognized that delta wave activity is diminished when the eyes are open. However, the findings of this study suggest that delta waves can also reflect changes in subject states to a certain degree. This assertion is supported by the use of ICA to eliminate electromyographic and oculomotor noise, potentially influencing the observed effects. Furthermore, the isolated analysis of the delta wave through filtering techniques underscores the sensitivity of this frequency band. Such findings could be pivotal for future EEG studies focusing on COVID-19 patients, particularly for elucidating alterations in brain activity. Previous research has associated low-frequency energy with long-range communication across brain regions [57]. The modifications in low-frequency activity observed in this study may indicate a substantial impact of the infection on the nervous system. Moreover, the results concerning complexity and entropy imply an increase in the chaotic nature of the neural system post-infection. Although none of the participants in this study was clinically diagnosed with “brain fog,” the EEG changes noted bear resemblance to those associated with “brain fog,” hinting at a potential underlying neurological impact of the infection [58].

Results indicate a gradation in susceptibility to cognitive impacts post-SARS-CoV-2 infection across different age groups. The most substantial cognitive changes were observed in young adults, a demographic that is not typically considered at high risk for severe COVID-19 implications. While previous studies have also shown that infection has a greater impact on young adults [59], the results of the present study provide additional evidence at the electrophysiological level for this conclusion. Warranting further investigates the long-term consequences of SARS-CoV-2 in younger populations. Notably, children also showed significant changes in HC and HM parameters, but this may be related to their rapid neurological development.

While this study has made discoveries regarding the impact of the coronavirus on the nervous system, it is not without its limitations and shortcomings. We endeavored to include as broad a population as possible, yet our study did not encompass all age groups, particularly the elderly. This omission means that the effects of the coronavirus on the neurological systems of older individuals remain unknown, given that some studies suggest this demographic may be more susceptible to such impacts [60]. Furthermore, our research did not involve continuous longitudinal tracking of the infected population, omitting long-term comparative data. The acquisition of such longitudinal information would be highly valuable and meaningful for understanding the full spectrum of the virus’s impact over time.

## Conclusions

In essence, this research furthers the existing knowledge on the neurological implications of SARS-CoV-2, underscoring the urgent requirement for a more profound understanding of the virus’s enduring effects on cognition. Particularly, it focuses on its impact on younger demographics, encompassing children and adolescents. The results intimate that the influence of SARS-CoV-2 is amplified within the younger populace. Although children and adolescents were relatively less affected, they exhibited noteworthy neurophysiological markers of abnormality, suggesting possible risk. This study, therefore, serves as a groundwork for more extensive research into potential therapeutic interventions and strategies to alleviate these cognitive alterations.

## Data Availability

This study constitutes a collaborative effort with BRAINNEWLIFE for data acquisition. As per the stipulations outlined in the agreement, the authors of this paper are bound by the obligation to safeguard the confidentiality of the underlying dataset, particularly the confidential and sensitive information embedded within. Nonetheless, all pertinent data metrics employed for the analysis explicated in this paper are delineated within the manuscript. Consequently, these metrics are readily available for replication and in-depth scrutiny. For researchers seeking access to pertinent anonymized data, we encourage the submission of formal requests to the corresponding author via email.

## Abbreviations

ADHD: Attention deficit hyperactivity disorder
DC: Direct current
dDTF: Direct directed transfer function
DTF: Directional transfer function
EEG: Electroencephalography
HA: Hjorth activity
HC: Hjorth complexity
HM: Hjorth mobility
ICA: Independent component analysis
IF: Instrumental frequency
MCI: Mild cognitive impairment
PDC: Partially directional coherence
